# Supplementary material for: Are carbon emissions trading and green financial instruments synergistic? -Comprehensive quantitative research based on content analysis
Source: PLoS One. 2024 Mar 7;19(3):e0298601. doi: 10.1371/journal.pone.0298601 (PMC10919601; doi:10.1371/journal.pone.0298601)
Supplement: S1 Data — (ZIP) [file pone.0298601.s001.zip › Data/地级市Z_F工作报告.zip!/╣≤╓▌/░▓╦│╩╨/└┤╫╘╣1⁄2╓┌║┼í╛┬φ┐╦╩2╛▌═°í┐_2014.docx]

各位代表：

　　现在，我代表市人民政府向大会作工作报告，请予审议，并请市政协委员和其他列席人员提出意见。

　　一、2013年工作回顾

　　刚刚过去的2013年，是全面贯彻落实党的十八大精神的开局之年，也是我市为与全省全国同步全面建成小康社会奋力拼搏的重要一年。在省委、省政府和市委的领导下，在市人大、市政协的监督、支持下，市人民政府团结和依靠全市各族人民，凝心聚力、攻坚克难，全力以赴抓发展、惠民生、促和谐，大力推进工业化、城镇化、农业现代化和旅游产业化“四化”同步发展，较好地完成了全年各项目标任务，迈出了赶超跨越的大步伐。

　　——全市经济保持快速发展。预计，全年实现地区生产总值427.85亿元，增长15.4%；完成全社会固定资产投资604亿元，增长51%；完成财政总收入70.25亿元（不含基金），增长15.5%，其中公共财政预算收入46.89亿元，增长29.2%；实现社会消费品零售总额111.2亿元，增长14%；金融机构存款余额 609.38亿元，贷款余额432.64亿元，分别比年初增长26.1%和23.5%。在全省经济发展增比进位综合测评预排位中，我市排名第5，比 2012年上升两位。

　　——经济结构不断调整优化。三次产业结构由2012年的14.5:37.3:48.2调整为 14.0:38.4:47.6。第一产业中粮经作物种植比由44:56调整为40:60；第二产业中规模以上轻重工业比由14.4:85.6调整为 18.5:81.5；第三产业中现代服务业快速发展。民营经济增加值占全市地区生产总值的比重达60%。

　　——“5个100工程”建设初见成效。共有10个产业园区、9个城市综合体、10个示范小城镇、8个现代高效农业示范园区和7个旅游景区纳入全省“5个100工程”，并全部完成规划编制，累计争取上级支持资金1.46亿元，启动实施建设项目370个，完成固定资产投资375.04亿元。

　　——人民生活水平持续稳定提升。城镇居民人均可支配收入18841元，农民人均纯收入5810元，增长14.2%；居民消费价格指数涨幅控制在2.1%以内；城镇登记失业率控制在4.2%以内；社会保障水平不断提高；以县为单位全面建设小康社会实现程度总体提高。

　　一年来，我们主要做了以下工作：

　　（一）狠抓项目建设，固定资产投资大幅提升

　　全年实施重大项目和重点工程412个，完成投资340亿元，增长97%。先后3次共组织82个重大项目集中开工，一批新项目、大项目启动建设。全年共获得中央和省转移支付资金104.13亿元，新增融资总额141.57亿元，落实建设用地2153公顷，为项目建设提供了有力保障。预计全市50万元以上固定资产投资完成410亿元，增长37.5%。

　　（二）狠抓园区建设，工业经济快速发展

　　预计全年完成工业投资180亿元，增长48.7%。全市产业园区完成投资218亿元，平坝夏云工业园、民用航空产业基地、西秀经济开发区等园区基础设施逐步完善，新建标准厂房 100.18万平方米，入驻率达90%。新增入园项目120余个，新增吸纳就业2.3万人。西秀工业园区列入省级新型工业化示范基地，镇宁轻工产业园被确定为“贵州省轻纺产业示范园”。工业项目建设加快，新增规模以上企业70户，晨春石材、顺立达纺织、华兴玻璃、汇景纸业等项目建成投产，安电三期、久联民爆、西南水泥等项目加快推进。预计全年实现全部工业增加值133.06亿元，增长15.3%，500万元以上工业增加值123亿元，增长20.3%。

　　（三）狠抓规划建设，城乡面貌持续改变

　　完成《安顺市城市总体规划（2009-2030）》大纲修编，中心城区控规覆盖率100%，各县区控规覆盖率85%以上，小城镇总规覆盖率100%。全市城镇化率达39.5%。违法用地、违法建设打击力度持续加大，控违拆违工作初见成效。全市城镇建设固定资产投资完成240亿元，增长87%。北部新区、新火车站片区基础设施建设步伐加快，安普城市快速干道建成通车，二环路全线贯通，贵安城市快速干道开工建设。市体育中心等项目建设进展顺利。燕安·国际佳缘、多彩万象旅游城等城市综合体建设加速推进，13个省、市级示范小城镇“8个1”和“8+3”工程全部开工。全市城镇供水普及率、县城以上污水处理率、生活垃圾无害化处理率分别达到95.9%、78.7%、72.6%，中心城区建成区人均公共绿地面积达3.5平方米。“四创”工作逐步推进，城市管理水平不断提升。“四在农家·美丽乡村”创建活动全面启动，西秀区鲍屯村等四个村寨列入全国美丽乡村创建试点。

　　（四）狠抓结构调整，农业生产提质增效

　　克服高温干旱灾害影响，粮食总产量达到63.13万吨，蔬菜、茶叶、中药材、精品水果种植面积分别达到109.94万亩、36.6万亩、23万亩、 35.11万亩，收购烤烟25.54万担。生态畜牧业加快发展，预计全年实现畜牧渔业增加值28.5亿元，占农林牧渔业总产值比重达47.9%。普定循环农业示范园、紫云低热河谷蔬菜产业示范园等园区发展迅速，示范带动作用逐步显现，全市省级农业产业示范园区实现总产值12亿元，带动6.3万人就业。

　　（五）狠抓旅游产业发展，第三产业逐步成长

　　不断完善旅游景区基础设施建设和配套服务，全年完成旅游业固定资产投资25.44亿元，增长58%。打造黄果树旅游升级版，实施黄果树景区“半边街”覆土绿化工程。国际佳缘、葡华等高星级酒店建设顺利推进。腾讯地图·智慧旅游安顺体验平台上线运行，编印完成首张《安顺旅游交通地图》。成功举办黄果树瀑布节、油菜花旅游节、中国格凸国际攀岩节、坝陵河低空跳伞挑战赛、镇宁半程马拉松等节庆活动和国际赛事。全年接待游客2805万人次，增长25.2%，实现旅游总收入249.07亿元，增长27.7%。交通物流、邮政通信、金融保险、商业零售、信息服务等服务业快速发展。

　　（六）狠抓基础设施建设，发展条件不断改善

　　完成交通建设投资72亿元，增长5.8%。沪昆高铁客专安顺段及沿线三个客运站点建设进度加快，安顺至六盘水铁路前期工作稳步推进。惠水至兴仁、六枝至镇宁高速公路及龙宫延伸段建成通车，普定至织金高速公路开工建设。全年新增高速公路通车里程47公里，实施县乡道改造134公里，开工建设通村油路 1200公里，两城区客运站搬迁工作全面完成。新增开通安顺至广州往返航线，黄果树机场全年进出港旅客达到6万人次。完成水利建设投资17.15亿元，增长53%。启动编制全市水资源综合利用规划，黔中水利枢纽工程加快推进，“引千入虹”工程隧洞全线贯通，石朱桥、新场河、纳井田水库建设进展顺利，新增有效灌溉面积2.04万亩。中缅油气管道安顺段投入试运行。完成电网建设投资4.76亿元。“三网融合”试点工作启动实施，城市宽带光纤网络改造步伐加快，全市3G移动电话用户达48.26万户。西秀区列为国家首批信息消费试点城市。

　　（七）狠抓生态建设，环境保护稳步推进

　　完成石漠化综合治理75.5平方公里、水土流失综合治理150平方公里，完成营造林35万亩，全市森林覆盖率达到42%。启动两城区山体公园化绿地建设工程，邢江河国家湿地公园规划通过国家评审。中心城区空气质量优良率保持100%，集中式饮用水源地水质达标率100%。新建11个污水处理厂投入运行，全市日处理污水能力达12.3万吨。医疗废弃物处置中心投入试运行，完成各县城垃圾填埋场建设。引导和支持企业加快节能技改，淘汰落后产能97.84万吨，万元地区生产总值能耗下降3.16%，主要污染物减排指标完成目标任务。

　　（八）狠抓招商引资，改革开放步伐加快

　　大力营造“亲商、安商、富商、敬商”的良好投资环境，对438项市级行政许可和155项非行政许可审批事项进行清理，取消15项，下放79项，便民服务事项办理时限平均减少11.3个工作日。积极组织参与泛珠会、酒博会等系列招商活动，先后赴香港、北京、广州等地举行招商引资推介活动，挂牌成立市投促局西南分局。全市招商引资实际到位资金770亿元，增长65.3%，其中省外境内项目实际到位资金580亿元，增长100%。与青岛开展新一轮对口帮扶合作，建立市政府驻青岛联络处，全市有24家部门、县区签订对口帮扶协议，合作建设青岛工业园。镇宁自治县与诸暨市结为友好县市，共建轻纺产业园区。

　　金融业加快发展，贵阳银行安顺分行、安顺农村商业银行挂牌开业，新增村镇银行3家，银行业金融机构营业网点达305个。组建安顺投资有限公司，改制成立市物资集团公司。市国资公司与国家开发银行贵州省分行共同搭建“四台一会”融资平台，中小企业融资扶持力度不断加大。“民营经济三年倍增计划”取得明显成效，新增规模以上民营企业50家，扶持微型企业2400户，民营经济增加值实现256亿元。积极探索城镇基层管理体制改革，试点设立社区服务中心并逐步推广。6个乡镇完成“乡改镇”、“镇改办”，“并村”工作顺利推进。第九届村（居）两委换届工作平稳有序推进。

　　（九）狠抓十件实事，社会事业全面发展

　　为民办十件实事全面完成：建成通村油路613.8公里；完成扶贫生态移民2874户12000人，建成保障性住房23717套，改造农村危房17239 户；完成5.6万户广播电视“户户通”工程并投入使用；解决农村20.2万人安全饮水问题，中心城区供水二期工程建成通水；减少农村扶贫对象10.93万人；城乡最低生活保障标准提高11%以上；全市城镇新增就业91578人，新增创业户2597户，创业带动就业12551人；完成113所农村中小学食堂标准化建设、43所农村中小学学生宿舍建设，完成33所乡镇公办幼儿园建设，实现乡镇幼儿园全覆盖；新型农村合作医疗参合率99.12%，实现全覆盖，完成市人民医院和市儿童医院门诊楼主体工程建设；中心城区新增150辆出租汽车上线营运，新增2242个公共停车位，建成5座人行天桥。

　　压缩行政经费的5%用于发展教育，启动实施教育“9+3”计划，加快推进230个“四项突破工程”项目建设，安顺职业技术学院新校区开工建设，西秀高中、平坝一中、关岭民中、普定职中、安顺旅游学校新校区投入使用。成功承办第十五届中国科协年会卫星会议，通过全国科技进步先进市考核验收。加强专业人才队伍引进和建设，与重庆大学签订战略合作协议。文化事业和文化产业加快发展，苗族英雄史诗《亚鲁王》荣获第十一届中国民间文艺“山花奖”，舞蹈《山尖尖》荣获第九届中国舞蹈“荷花奖”银奖。社会救助体系不断完善，继续实施农村低保季节性缺粮户粮食救助制度，市残疾人康复中心开工建设。全市人口出生率控制在 12.4％。以内、自然增长率5.7％。。安全生产监管力度不断加大，事故起数和死亡人数实现“双降”。切实加强基层矛盾化解工作，认真落实领导包案督导化解重大突出矛盾和信访问题制度。深入推进“平安安顺”建设，群众安全感指数93.63%，从2012年的全省第8位上升到第6位。国防教育、民族、宗教、双拥、档案、史志、外事、侨务、对台、人防、慈善、气象、水文、防震减灾、质量技术监督、粮食安全保障、住房公积金管理等工作取得新成绩。

各位代表，一年来，我们切实加强政府自身建设，提高政府服务发展能力。预防处置突发公共事件的能力不断提高，应急管理工作进一步加强。电子政务全面推行，政府门户网站改版升级。市、县两级政务服务中心效能提高，市公共资源交易中心运行良好。加强廉政建设和行政监察、审计监督，切实推进干部作风转变，全市上下干事创业的氛围更加浓厚。自觉接受人大及其常委会的法律监督和工作监督，自觉接受政协的民主监督，办理人大建议69件、政协提案179件，满意率 100%。认真听取各民主党派、工商联和无党派人士的意见建议，积极支持工会、共青团、妇联、文联、科协、残联、侨联、红十字会、关工委等人民团体依法按章履行职责。

　　回顾过去一年的工作，我们深切体会到，在国内外经济下行压力加大的情况下，能够实现持续快速发展、排名增比进位，成绩来之不易，这是省委、省政府和市委坚强领导，市人大依法监督、市政协民主监督与支持的结果，是全市广大干部群众及社会各界同心同德、艰苦奋斗的结果。在此，我谨代表市人民政府，向全市各族人民，向各位代表、政协委员，向各民主党派、工商联、无党派人士、人民团体，向驻安人民解放军、武警消防官兵、公安政法干警、中央和省驻安单位，向老同志和所有关心、支持我市改革开放及现代化建设的各界人士，表示衷心的感谢并致以崇高的敬意！

　　在看到成绩的同时，我们也清醒地认识到存在的问题和不足，主要表现在：一是经济总量小、人均水平低、发展速度慢等问题没有得到根本改变，个别指标未完成年初目标任务；二是与省内其它市州的快速发展相比，我市部分主要经济指标仍然存在不小差距；三是工业不强、城镇不优、农业偏弱、旅游产业滞后，产业结构不合理、链条不长，缺乏集群发展能力；四是少数地方、部门服务意识不强，“庸、懒、散、慢、浮”的现象不同程度存在，招商安商环境还不优；五是思想不够解放，观念陈旧，思路不宽，抓发展的办法不多，创造力、执行力亟需进一步提高，等等。为此，我们将正视存在的问题，不争论不议论，不懈怠不折腾，提振精气神，树立自信心，咬定目标不放松，千方百计提速度，通过更好更快发展来解决我们前进中遇到的困难和问题。

　　二、2014年的重点工作

　　 2014年，市人民政府坚持以党的十八大精神为指引，全面贯彻落实十八届二中、三中全会，省委十一届四次全会和市委三届五次全会精神，坚持主基调、主战略，紧紧围绕提速发展、奋力赶超、同步小康的总目标，按照“稳中求进、进中求快、改革创新”的总要求，始终把全面深化改革贯穿于经济社会发展各个领域各个环节，全力推进工业化、城镇化、农业现代化和旅游产业化“四化”同步发展，努力实现全市经济社会持续健康稳定发展。

　　2014年，全市经济社会发展的主要目标是：地区生产总值增长18%，突破500亿元；全社会固定资产投资增长65%，突破1000亿元；公共财政预算收入增长 25％；500万元以上工业增加值增长24%以上；旅游总收入增长28%；社会消费品零售总额增长16%；城镇居民人均可支配收入增长18%；农民人均纯收入增长20%；人口自然增长率控制在5.7％。以内；居民消费价格指数涨幅控制在3.5%左右；完成国家和省下达的节能减排任务；全力做好第三次全国经济普查工作。

　　围绕实现上述目标，重点从以下四个方面抓好落实。

　　（一）凝心聚力抓发展，努力做大经济总量，促进后发赶超

　　发展是解决所有问题的关键，我们只有增强发展自信，激活内生动力，做大经济总量，才能提升综合实力，改变落后面貌，实现后发赶超。

　　一是全面做大固定资产投资规模，着力在项目建设上有新突破

　　超前统筹谋划项目。充分释放国发〔2012〕2号、黔党发〔2012〕15号文件能量，围绕“5个100工程”，高水准、超常规谋划一批产业链长、带动性强的大项目、好项目，提高亿元以上项目比重，以大投资带动大发展。加强储备项目要件手续完善工作，切实提高项目成熟度。市级继续安排5000万元用于项目前期工作，确保重大项目和重点工程顺利实施。对重大项目和重点工程实行市级领导干部挂帮考评机制。确保50万元以上固定资产投资完成800亿元，增长 95%。

　　提速推进项目建设。认真抓好270个亿元以上重大项目和重点工程的责任分解、定期调度，对项目进度、开工竣工、投产达产进行追踪问效、督查考核，确保项目建设快速推进。拓宽政银企合作领域，确保贷款余额增长30%以上，建立政府性资金存款按金融机构贡献度分配机制，规范完善政府性债务融资程序、资金管理和清偿机制，有效发挥国资公司、安投公司等投融资平台的作用，灵活运用多种金融工具提高直接融资比重。通过盘活存量、争取增量最大限度保障建设用地需求，推动农村经营性集体建设用地在符合规划的前提下进入市场，探索建立城乡统一的建设用地市场。依法严厉打击违法阻工、强揽工程等行为，维护良好的建设环境。

　　强化基础设施建设。全面实施全市综合交通建设“三年攻坚行动”方案，市级安排交通项目建设专项资金2000万元，全年完成交通基础设施建设投资150亿元。配合做好沪昆高铁客专、安顺至六盘水铁路安顺段建设工作。开工建设贵阳花溪至安顺、安顺至紫云高速公路，加快推进普定至织金高速公路建设。实施国省干线公路提等改造234公里，加快镇宁至坝草、紫云至镇宁公路提等改造建设，完成沪昆高速公路安顺境内11个收费站改扩建工程。加强黄果树机场建设，加大航线培育力度，力争开通安顺至上海、安顺至青岛航线。建成水上搜救指挥中心，完成坝草码头改造工程。全面开展骨干水源工程、引提灌工程和地下水利用工程“三大会战”，完成水利投资26亿元，力争开工建设黄家湾水利枢纽工程，协助推进黔中水利枢纽工程建设。实施主干电网升级工程，加快城乡电网建设和改造，完成电网投资5.58亿元。逐步扩大“三网融合”试点范围，实现3G网络全覆盖，推进4G网络中心城区全覆盖和普定县试点工作，完成通讯投资7.8亿元。

　　二是全力加快工业经济发展，着力在产业园区建设上有新突破

　　加大工业投资力度。多渠道筹集工业和信息化发展专项资金1亿元，安排以奖代补专项资金2000万元，以“九大行动计划”为抓手，培育壮大航空、特色轻工、原材料、能源和战略性新兴产业“五大支柱产业”，努力将建筑业、运输业培育成为重要支柱产业。坚持存量扩能、增量提速，推进煤电铝一体化，促进工业项目扩能技改，实现资源就地就近转化和产业效益最大化。开工建设安电三期、华润水泥，加快推进久联民爆、恒远建材、中铝装备、联鑫工艺等工业项目建设，推动石材产业加快发展，建立西南国际石材交易中心，全年完成工业投资330亿元。

　　加强工业运行调度。抓好煤、电、油、运等要素调度，加快煤炭产业兼并重组，确保全年煤炭产量1200万吨、发电量85亿千瓦时以上。认真开展“企业服务年”活动，强化“一企一策”、“一事一议”精细化服务。深化与清华大学、重庆大学等高校的合作，推进产学研一体化，促进科研成果转化为经济成果。完成全部工业增加值171亿元，增长24%，500万元以上工业增加值161亿元，增长 24%以上。

　　加快工业园区建设。不断完善园区道路、供电、供水、通讯、污水处理、垃圾处理等配套基础设施，有序推进标准厂房建设，制定工业园区管理办法，推广“产业园区+标准厂房+职业教育”模式，不断提升园区发展的承载力。促进军民融合深度发展，支持贵飞公司打造百亿级企业，推动安顺国际飞机制造公司、贵州云马汽车公司等航空和装备制造企业做大做强，支持军工企业人才创办、领办科技型企业。加大与对口帮扶城市的协作力度，确保青岛-安顺产业园建设取得实效，加快民用航空产业基地、西秀经济开发区等重点园区建设，培育百亿级、千亿级园区，产业园区工业增加值占全部工业增加值比重达到 65%以上。

　　严格推进节能减排。抓好重点行业、重点企业的节能管理和技术改造，逐步淘汰落后产能，引导和支持企业调整产品结构，降低能耗和生产成本，确保万元地区生产总值能耗降低3.2%。推动传统产业转型升级，形成有利于资源节约和环境保护的产业体系。

　　三是全力推进城镇化进程，着力在城市管理上有新突破

　　加快城市规划建设。完成中心城区城市总体规划修改工作，加大控规覆盖密度，全市控规覆盖率达90%以上。加快路网及沿线规划建设，扩大城市规模，贵安城市快速干道、二环路建成通车。快速推进旧城改造和新区建设，提升城市形象，积极筹集城市建设资金，做大做强中心城区，全力推进南出口和东出口棚户区、中华西路棚户区二期、华西商住片区、汪家山城中村、历史文化街区改造，加快北部新区、新火车站片区建设。推动重点城建项目建设，不断完善城市功能，全面完成 “引千入虹”工程，推进虹山湖景观整治二期，实施贯城河河道治理及沿线景观整治；加快城市综合体建设步伐，打造城市新地标，加大市体育中心、市大剧院、市档案馆建设力度，力争启动老年活动中心和青少年活动中心改造建设。

　　加大城镇建设力度。筹集城镇化建设市级专项资金4000万元，安排以奖代补专项资金2000万元，完成城镇建设固定资产投资360亿元。加快省、市级示范小城镇建设，基本建成“8个1”和“8+3”工程。全力承办好全省第三届小城镇建设发展大会，市级安排资金1.5亿元，因势利导、因地制宜，培育发展一批交通枢纽型、旅游景观型、绿色产业型、工矿园区型、商贸集散型等特色示范小城镇。全市城镇化率达到41%。

　　深入推进城市管理。发挥职能部门作用和街道社区自治及服务功能，整合力量开展城市联合执法，充分调动全社会力量共同参与，大力治理不文明行为，不断加大控违拆违力度，提升城市精细化管理水平。加强城市交通管理，有效缓解城区拥堵问题。继续推进绿化、美化、净化、亮化工程，实施中心城区山体公园化绿地建设，实现中心城区建成区绿地率30%以上。加大“四创”工作力度，年内申报全省文明城市，争取获全国文明城市提名。大力创建双拥模范城（县）。

　　四是大力发展第三产业，着力在打造旅游升级版上有新突破

　　加快旅游产业发展升级。全力打造世界知名、国内一流的旅游目的地和休闲度假胜地，出台加快全市旅游业发展的意见，市级筹集1250万元专项资金，安排2000万元以奖代补专项资金支持旅游产业发展升级，举办全市首届旅游发展大会。加大旅游产品设计开发力度，不断推动旅游与文化、体育等深度融合发展，拓展旅游业态，构建“快进慢出”的旅游发展新格局。充分结合示范小城镇、美丽乡村建设，大力发展乡村旅游。设计开发特色鲜明、广受市场欢迎的旅游商品，培育扶持一批特色旅游商品生产经营企业。强化宣传推介和市场营销，大力开拓国内外旅游市场，不断提升安顺旅游对外知名度和影响力，全年接待游客3590万人次以上，增长28%，旅游总收入319亿元以上，增长28%。

　　强化景区龙头带动作用。切实发挥5A级景区龙头示范带动作用，加快大屯堡文化旅游区建设，构建大黄果树旅游圈。支持兴伟石博园、多彩万象旅游城等申报4A级旅游景区。积极引进战略投资者参与景区建设，加大旅游商品市场、景区停车场等基础设施建设力度，加强景区精细化管理，加快黄果树、龙宫旅游升级版建设步伐。不断提升黄果树瀑布节、油菜花旅游节、中国格凸国际攀岩节、坝陵河低空跳伞挑战赛、镇宁全程马拉松等节庆赛事活动规模和档次，举办孔子文化节，打造对外交流合作的重要平台。

　　丰富优秀旅游城市内涵。加快推进市游客集散中心等基础设施建设，开通旅游景区专线、城市观光巴士，加强旅游信息网络平台建设。围绕文庙、王若飞故居等标志性景点，充分发掘安顺历史文化资源，提升城市旅游品位。完成2个以上五星级酒店建设，引导建设一批度假型、商务型和特色主题酒店，完善餐饮、住宿、购物、娱乐等要素。加快体育运动公园项目建设步伐。积极争取纳入入境游客落地签证范围。探索建立旅游环境考核考评奖惩机制，不断提升城市旅游服务能力和水平。

　　推动现代服务业加快发展。合理规划建设商贸、建材、汽车、农产品等市场体系布局，打造一批特色鲜明的商业街区。启动金融城建设工作。加快黄桶-幺铺物流园区建设，全面建成投用黔中农产品交易大市场、西秀经济开发区农产品批发市场。做优做强旅行社，加强服务行业培训管理，开展酒店、导游、出租车司机等“十佳”评选活动，推动服务业发展迈上新台阶。

　　五是做大做强对外开放型经济，着力在招商引资上有新突破

　　创新招商引资方式。坚持引进来和走出去相结合，重点围绕“5个100工程”建设，创新招商方式，提高招商引资的针对性和实效性。坚持招大商、选好商、引大资，实施好“央企入安”、“民企入安”和“外企入安” 专项行动，主动与世界500强、中国500强企业和其他大集团、大企业对接，引导更多的战略投资者落户安顺。市级安排招商引资工作和奖励经费1100万元，精心筹备组织好赴外开展的各种招商推介会，积极参加省内外大型招商引资活动，提高招商项目成功率，实现招商引资实际到位资金突破1000亿元。

　　积极扩大对内对外开放。深化区域合作，加快融入长江经济带、泛珠三角、成渝经济区发展，扩大与长三角、环渤海、港澳台等区域合作。加强与对口帮扶城市的全方位合作，组建青岛招商引资分局。争取各民主党派中央大力支持。实施提高民营经济比重五年行动计划，新增规模以上民营企业50家，扶持微型企业2000 户，民营经济增加值占地区生产总值的比重达63%。确保实际利用外资6000万美元，实现进出口总额3300万美元。

　　切实优化投资环境。深化行政审批制度改革，优化流程，简化环节，把我市打造成为全省行政审批事项最少的地方之一。加强投资项目全程跟踪协调服务，进一步完善并联办理和代办服务制，提高项目履约率、入园率、资金到位率、投产达产率。加快各级政务服务中心规范化建设，建立投资环境量化评估体系，严肃查处影响投资环境的行为，努力营造公开、公平、公正的投资环境。

　　（二）围绕“三农”抓增收，奋力扶贫攻坚，推进小康目标建设

　　全面建成小康社会，重点在农村，关键在农民，难点在破除城乡二元结构。我们必须把服务“三农”作为工作的重中之重，把扶贫攻坚作为“第一民生工程”，向贫困发起“总攻”，为与全省全国同步全面建成小康社会打下坚实基础。

　　一是扎实推进农业现代化，着力在结构调整上有新突破

　　突出抓好农业产业结构调整。在确保粮食总产量65万吨的前提下，力争粮经作物种植比调整到34∶66。种植蔬菜120万亩、水果49.2万亩、中药材 36万亩，新增茶园7万亩，收购烤烟22万担。大力发展生态畜牧业，实现肉蛋产量18万吨，水产品产量1.4万吨，畜牧渔业产值增长10%。统筹农业现代化发展专项资金4827万元，安排以奖代补专项资金2000万元，召开全市农业产业结构调整现场观摩会，大力推动农业规模化、标准化、产业化发展，让农业成为有奔头的产业。

　　加快建设现代高效农业示范园区。围绕沪昆高速公路沿线和省市级现代高效农业示范园区，打造我市农业名片和观光农业示范带。启动建设青岛-安顺共建现代高效农业示范园区。加快全国丘陵山地农业机械化示范区建设，全市农机总动力达到185万千瓦，农业机械化综合水平达 45%。

　　促进农民增收。加快农村“三权”确权颁证工作，建立农村产权交易市场，有序推进农村土地合理流转，破解农村融资难的瓶颈，有效增加农民财产性收入。积极推进新型农业经营体系建设，加大对合作社、家庭农场的扶持力度。加强农村集体资金、资产、资源“三资”管理，壮大农村集体经济实力。建立健全龙头企业与农民之间的利益连接机制，大力发展农村农业社会化合作组织，实现各类农村专业化合作组织突破1000个，提高农业经营效益，促进农民增收，让农民成为体面的职业。

　　二是扎实加大扶贫攻坚力度，着力在扶贫成效上有新突破

　　大力推进扶贫工作。积极探索扶贫开发工作新办法新途径，改变对贫困县的考核考评机制，按照“县为单位、动态管理、规模控制、分级负责、精准识别”的原则，逐村逐户制定帮扶措施和脱贫目标，健全对象识别“瞄准”机制，做到结对帮扶、产业扶持、教育培训、农村危房改造、扶贫生态移民、基础设施“六个到村到户”。深入实施滇桂黔石漠化片区区域发展与扶贫攻坚工程。不断加强整县推进工作，确保普定县和9个乡镇实现“减贫摘帽”。继续实施扶贫生态移民工程，让农民搬得出、稳得住、能致富。认真开展“雨露计划”等各项工作，培训2.6万人，不断提高农民生产技能。

　　加大扶贫资金投入。力争获得中央、省级扶贫资金突破3亿元，积极争取信贷扶贫资金，逐步增加市级扶贫资金。落实好市政府与省扶贫办的合作协议，用好省扶贫办专项资金，抓好共建黔中山地高效蔬菜产业化扶贫示范区工作。加大产业扶贫投入，大力培育贫困地区最有条件发展的产业，农村基础设施建设、农村公共卫生项目及资金方面进一步向贫困地区倾斜。加强扶贫、涉农资金审计监督，提高资金使用效率。

　　三是扎实加快新农村建设，着力在“四在农家·美丽乡村”工作上有新突破

　　加快农村基础设施建设。整合各类涉农资金，加强农田水利、机耕道等农业设施建设。完成1130公里通村油路（水泥路）建设任务，西秀区、平坝县、开发区提前实现村村通油路（水泥路）。开工建设偏坡、大洞口、木拱河等水库，治理中小河流12条，完成病险水库治理75座，推进安西灌溉配套改造工程，新增灌溉面积6.5万亩。

　　加快农村小康建设。以“四在农家·美丽乡村”创建活动为抓手，市级投入创建资金2000万元，按照“一建四改治八乱，五有四化三提高”的要求，创建一批示范村寨，高标准开展新农村建设。大力实施小康路、小康水、小康房、小康电、小康讯和小康寨农村基础设施建设六项行动计划，推动交通、通讯网络、环境卫生等基础设施及公共服务向农村延伸，让农村成为安居乐业的美丽家园。

　　四是扎实推进生态环境建设，着力在农村环境整治上有新突破

　　强化农村环境改善。以创建生态示范乡、村为抓手，大力开展农村环境综合整治，加强农业面源污染治理，有效改善农村生产生活条件。逐步推进农村生活垃圾 “村收集、乡清运、县处理”。严格执行饮用水源保护区管理规定，强化夜郎湖等集中式饮用水源地环境保护，确保水质全面达标。规范矿山环境治理保证金缴存、使用和监管，积极实施地质灾害和矿山环境恢复治理工程。

　　强化生态环境保护。继续抓好退耕还林、天然林保护二期、珠江防护林体系建设等重点林业生态工程，完成营造林36万亩，完成石漠化综合治理92.48平方公里，治理水土流失150平方公里，确保森林覆盖率达43.5%以上。启动建设邢江河国家湿地公园。坚持开发与保护并重，建立完善生态补偿激励机制，守住生态底线，切实保护好青山绿水。

　　（三）关注民生事业，创新社会治理，建设和谐美丽新安顺

　　我们要着眼于维护最广大人民的根本利益，有效解决人民群众最关心、最直接、最现实的利益问题，创新社会治理，最大限度增加和谐因素，增强发展活力，建设和谐美丽新安顺。

　　一是全力提高人民群众生活水平，着力在发展民生上有新突破

　　提升就业和社会保障水平。健全完善城乡公共就业服务体系，大力推进创业型城市创建工作，城镇登记失业率控制在4.2%以内。积极构建和谐劳动关系，切实维护劳动者的合法权益。不断提高新农合参合率和养老保险覆盖率，加快推进覆盖城乡居民的社会保障体系建设。继续抓好“保供稳价”工作和“菜篮子工程”，确保居民消费价格保持总体稳定。

　　全面推进社会事业。推动混合所有制经济融入社会事业各领域，全市教育、卫生、文化、体育固定资产投资达到 32.5亿元。深入实施教育“9+3”计划和“四项突破工程”，鼓励吸引社会力量兴办教育，推进非义务教育阶段混合所有制、多元化主体办学，科学规划城乡校点布局，缩小区域内校际差距，大力支持安顺学院通过教育部本科教学水平合格评估，加快安顺职业技术学院新校区建设，支持有条件的中学申报省级示范性高中，制定完善职称、待遇等向农村学校和教学一线教师倾斜的措施办法。大力培养和引进重点领域、重点行业专业技术人才，市级安排2600万元人才发展专项资金，建立完善人才管理机制，营造富有活力的人才环境。深化医药卫生体制改革，加快县级公立医院改革步伐，鼓励社会办医，年内完成市人民医院主体工程和市儿童医院新院建设。加强人口计生工作，确保实现“双降”目标。推动文化事业和文化产业加快发展，精心打造一批独具特色的文化精品，形成一批具有市场影响力和竞争力的文化品牌。

　　提高城乡公共服务水平。优先发展公共交通，实现城郊结合部公交全覆盖，所有县城城区开通公交车。开工建设市客运枢纽中心，推进各县城客运站建设，开工建设乡镇客运站16个，建成农村公路招呼站900个。有效提升城乡公共服务设施建、管、养水平。深入推进广播电视村村通、户户通、电影放映、农家书屋、文化家园等工程。

　　二是全力维护社会和谐稳定，着力在加强和创新社会治理上有新突破

　　深化“平安安顺”建设，强化社会治安综合治理，依法严密防范和惩治各类违法犯罪活动,加强社区矫正工作，加快推进强制戒毒所、工读学校和特殊人群康复基地建设，进一步提高人民群众的安全感和满意度。继续实施“三关爱工程”，加强对流浪儿童、乞讨人员救助管理，重视失独家庭养老扶助，健全残疾人社会保障服务体系。落实安全生产责任，强化安全生产监督管理，防止重特大安全事故的发生。加强食品药品安全监管，保障农产品质量安全。健全重大决策社会稳定风险评估机制，增强应急处置能力，健全完善网络突发事件应对处置机制，加快建设市级救灾物资储备库、粮油储备库。总结推广“上关经验”，学习和借鉴“枫桥经验”，构建畅通有序的诉求表达、心理干预、矛盾调处、权益保障机制，建立涉法涉诉信访依法终结制度。重视国防后备力量建设，支持军队、武警部队建设和法院、检察院工作。加强国家安全、司法行政、宗教、外事、侨务、对台、人防、档案、史志、气象等工作。

　　（四）实事实干抓落实，深化改革求发展，取信于民见成效

　　安顺正处在一个搞建设、干事业的大好时期，我们既要拿出敢干、实干的胆略和勇气，又要汇聚能干、会干的智慧和能力，真抓实干，改革创新，务求实效。

　　一是扎实做好十件实事，着力在利民惠民上有新突破

　　⑴新建保障性住房2.8万套；改造农村危房1.5万户；启动全市棚户区改造18191户。⑵中心城区新增4000个公共停车位、200辆出租车，新建 12所公厕，修通10条断头路，启动搬迁民主路夜市。⑶完成1.1万人扶贫生态移民搬迁；减少农村扶贫对象10万人；完成农村10万人安全饮水工程。⑷城镇居民医保和新型农村合作医疗政府补助标准提高到320元；城镇居民养老保险参保人数92万人，60岁以上25万人；失独家庭特别扶助金每人每年达到 4800元。⑸完成广播电视“村村通”2.5万户建设项目，放映农村公益电影22000场。⑹城乡最低生活保障标准在2013年基础上提高15%以上；农村敬老院实现乡镇全覆盖；新建农村留守儿童之家8个以上。⑺全市完成城镇新增就业46000人，新增创业户1560户，创业带动就业6240人。⑻实施中心城区山体公园化绿地建设工程，完成山体公园建设2个，小型游园建设7个，新增公共绿地130万平方米。⑼人口计生利益导向“四项制度”覆盖目标人群10 万人（户），计生“三结合”帮扶农村计生家庭1万户，新建乡级计生服务站3个、村级计生服务室50个，对1.23万对计划怀孕夫妇实施免费孕前优生健康检查。⑽扩大公交线路覆盖面，开通中心城区至镇宁自治县大山镇公交，开通中心城区至平坝县、普定县公交，开通双层环城观光公交；关岭自治县、紫云自治县县城开通公交。

　　二是深入推进各项改革，着力在重点领域上有新突破

　　全面贯彻落实《中共中央关于全面深化改革若干重大问题的决定》和省委、市委的实施意见，充分发挥安顺试验区载体作用，争做改革的先行者、行动派，抓出一批试点经验，加以推广，让改革发展成果更多更好更公平地惠及人民群众。

　　优化政府机构设置、职能配置、工作流程，完善决策权、执行权、监督权既相互制约又相互协调的行政运行机制。严格绩效管理，突出责任落实，确保权责一致。凡属事务性管理服务原则上都要引入竞争机制，通过各种方式向社会进行购买。充分发挥市场在资源配置中的决定性作用，深化投资体制改革，确立企业投资主体地位，按照市场化、产业化原则，积极引导各类战略投资者、各种经济成分入股国有企业，形成混合所有制企业。充分发挥县区在发展中的主动性和积极性，深入推进西秀区全国发展改革试点城市各项工作，做大做强中心城区，增强中心城区的辐射带动能力，帮助基础条件较差的贫困地区不断增强实力，迎头赶上，形成竞相发展、你追我赶的良好态势。加快平坝县、普定县“撤县设区”工作步伐，支持安顺经济技术开发区申报国家级开发区。积极稳妥推进乡镇行政区划调整，形成布局合理、服务高效的行政区划和行政管理体制。加快户籍制度改革，全面放开落户限制，农村居民自愿转为城镇居民，享有与城镇居民同等的社会保障待遇，保留农村集体经济组织成员的权益，享受各项惠农政策。

　　研究制定分类推进改革的实施方案，把改革的思想和要求变成可操作的行动计划和具体项目，用改革增动力促发展，向改革要红利惠民生。

　　三、以人民满意为标准，加强政府自身建设

　　各位代表，面对全省各地竞相发展的逼人态势，我们只有“干”字当头、“敢”字为先、“拼”字求胜、奋起直追，才不会落后掉队；我们只有打破常规、破釜沉舟、背水一战，才能再创安顺发展的新辉煌，切实做到对人民负责、为人民服务、让人民满意。

　　（一）解放思想，建设创新型政府。我们要把解放思想、锐意创新作为政府工作的根本动力，增强机遇意识、责任意识、发展意识，继承和发扬大胆创新、勇于探索、敢为人先的“顶云经验”，善于用改革的办法和创新的举措突破瓶颈制约，破解发展难题，不断提高推动发展的能力和水平。推动政府从“管理型”向“服务型”、从“封闭型”向“开放型”转变；推动干部从“观望型”向“实干型”、从“平庸型”向“进取型”转变。敢与强的比、敢与高的攀、敢同勇的争、敢跟快的赛，以更加开阔的视野和更加开放的心胸审视自己、倒逼自己，以抓铁有痕、踏石留印的魄力和勇气自加压力、奋力争先，激发全市人民干事创业的激情，树立安顺人敢干事、能干事、干成事的良好形象。

　　（二）提升能力，建设高效型政府。全面深化改革，不断加强政府能力建设，提高政府在公共服务、市场监管、社会管理、环境保护等方面的能力。加强领导干部学习教育，解决本领恐慌问题，切实提高破解复杂突出问题、处理公共突发危机、加快经济社会发展的能力。进一步理顺行政体制机制，最大限度减少政府对微观事务的管理，着力解决部门职能交叉、职责不清等问题。认真落实首问责任制、限时办结制、责任追究制，坚决纠正和严肃查处上有政策、下有对策，有令不行、有禁不止的行为。

　　（三）转变作风，建设服务型政府。认真开展党的群众路线教育实践活动，严格执行中央“八项规定”和省委“十项规定”，坚决反对“四风”，大兴求真务实之风，崇尚实干、力戒空谈，不断提高各级干部的执行力和落实力。进一步精简会议、文件，减少事务性应酬，真正把精力放在抓落实、谋发展上。大力倡导主动作为、雷厉风行、敢于担当的良好作风，整治“庸、懒、散、慢、浮”的行为，坚决纠正“不作为、慢作为、乱作为”的现象，努力做到“去庸求进、去懒求勤、去散求严、去慢求快、去浮求实”。牢固树立全心全意服务企业、服务基层、服务人民群众的理念，切实维护好、实现好、发展好人民群众的根本利益。

　　（四）依法行政，建设法治型政府。按照法定权限和程序行使职权、履行职责，切实做到依法决策、依法行政、依法管理。加强信息公开工作，回应社会关切，提高政府的公信力。主动接受人大及其常委会的法律监督、工作监督和政协的民主监督，认真办理人大建议和政协提案。充分听取各民主党派、工商联、无党派人士和人民团体的意见建议。强化监察、审计等内部监督，自觉接受舆论监督和社会监督。

　　（五）规范制度，建设廉洁型政府。按照“照镜子、正衣冠、洗洗澡、治治病”的要求，加强政府廉政建设。坚持用制度管权管事管人，把权力关进制度的笼子，让人民监督权力，让权力在阳光下运行。强化对重点领域行政权力运行的监督，推进“三公”经费、行政经费公开，干干净净做事，清清白白做人。加大领导干部经济责任审计力度，查处大案要案，遏制腐败行为，做到干部清正、政府清廉、政治清明。

　　各位代表，安顺正处在提速发展、奋力赶超、同步小康的关键时期，我们深感责任重大、使命光荣，唯有脚踏实地、扎实苦干、艰苦奋斗才能实现宏伟目标。让我们更加紧密地团结在以习近平同志为核心的党中央周围，在市委的坚强领导下，只争朝夕、攻坚克难，改革创新、锐意进取，全力建设实力安顺、宜居安顺、开放安顺、平安安顺，为创造安顺人民更加幸福美好的生活而努力奋斗！
